# In-Orbit MapAnything: An Enhanced Feed-Forward Metric Framework for 3D Reconstruction of Non-Cooperative Space Targets Under Complex Lighting

**DOI:** 10.3390/s26072026

**Published:** 2026-03-24

**Authors:** Yinxi Lu, Hongyuan Wang, Qianhao Ning, Ziyang Liu, Yunzhao Zang, Zhen Liao, Zhiqiang Yan

**Affiliations:** Research Center of Space Optical Engineering, Harbin Institute of Technology, Harbin 150001, China; 22b921002@stu.hit.edu.cn (Y.L.); fountainhy@hit.edu.cn (H.W.); ningqh@hit.edu.cn (Q.N.); meyondlzy@stu.hit.edu.cn (Z.L.); cloud26z@stu.hit.edu.cn (Y.Z.); 20b921003@stu.hit.edu.cn (Z.L.)

**Keywords:** 3D reconstruction, non-cooperative space targets, challenging illumination, feed-forward transformer, parameter-efficient fine-tuning, deep learning

## Abstract

Precise 3D reconstruction of non-cooperative space targets is a prerequisite for active debris removal and on-orbit servicing. However, this task is impeded by severe environmental challenges. Specifically, the limited dynamic range of visible light cameras leads to frequent overexposure or underexposure under extreme space lighting. Compounded by sparse textures and strong specular reflections, these factors significantly constrain reconstruction accuracy. While existing general-purpose feed-forward models such as MapAnything offer efficient inference, their geometric recovery capabilities degrade sharply when facing significant domain shifts. To address these issues, this paper proposes an enhanced 3D reconstruction framework tailored for the space environment named In-Orbit MapAnything. First, to mitigate data scarcity, we construct a high-quality space target dataset incorporating extreme illumination characteristics, which provides comprehensive auxiliary modalities including accurate camera poses and dense point clouds. Second, we propose the SatMap-Adapter module to mitigate feature degradation caused by severe specular reflections. This architecture employs a hierarchical cascade sampling strategy to align multi-level backbone features and utilizes a lightweight adaptive fusion module to dynamically integrate shallow photometric cues, intermediate structural information, and deep semantic features. Finally, we employ a weight-decomposed low-rank adaptation strategy to achieve parameter-efficient fine-tuning while strictly freezing the pre-trained backbone. Experimental results demonstrate that the proposed method decreases the absolute relative error and Chamfer distance by 15.23% and 20.02% respectively compared to the baseline MapAnything model, while maintaining a rapid inference speed. The proposed approach effectively suppresses reconstruction noise on metallic surfaces and recovers fine geometric structures, validating the effectiveness of our feature-enhanced framework in extreme space environments.

## 1. Introduction

With the increase in space activities and the proliferation of debris, close proximity on-orbit servicing has become increasingly critical. High-precision 3D reconstruction of non-cooperative targets is essential to the success of such missions. Due to the limitations of active imaging devices in terms of power consumption and operating range in space, passive multi-view visible-light-based reconstruction has emerged as a practical and viable approach.

**Figure 1:**
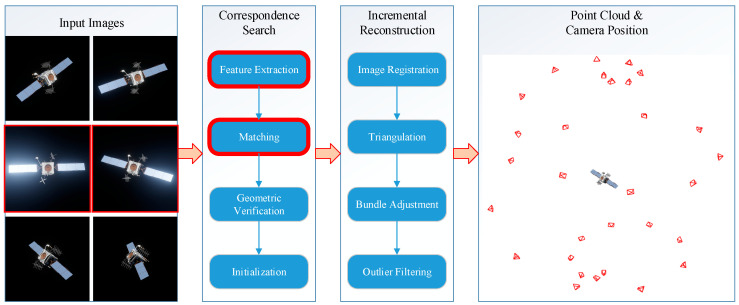
Schematic diagram of the SfM algorithm. The modules highlighted in red boxes are prone to failure in space environments.

Structure from Motion (SfM) has long served as the de facto standard for multi-view 3D reconstruction. As illustrated in Figure 1, this paradigm relies on a sequential pipeline comprising feature extraction, geometry estimation, and triangulation. However, despite its theoretical maturity, such a cascaded approach faces severe limitations when applied to non-cooperative space targets:

**Robustness Limitations Due to Feature Dependency:** Space target surfaces are often covered with multi-layer insulation (MLI), exhibiting sparse texture and strong specular reflection. This leads to unreliable feature extraction and matching, resulting in geometric holes and noise in the reconstruction.

**Cascading Error Propagation:** Errors from early stages (e.g., feature matching) accumulate through subsequent steps (e.g., triangulation, dense reconstruction) without the possibility of backward correction, lacking global consistency and closed-loop optimization.

**Computational Efficiency Bottleneck:** The multi-stage iterative optimization process is computationally intensive and time-consuming, making it difficult to meet the real-time requirements of on-orbit operations.

Therefore, urgent needs exist for accurate and efficient 3D reconstruction methods adaptable to extreme space lighting for autonomous on-orbit operations. Recently, Transformer-based architectures like DUSt3R and VGGT have shifted the paradigm by discarding cumbersome geometric constraints for unified inference. The state-of-the-art MapAnything further achieves a qualitative leap in versatility and efficiency via factorized scene representations. However, directly applying these general models to space environments causes severe “Domain Shift” due to extreme HDR lighting and unique material reflections, leading to marked performance degradation.

To address this, we propose an efficient reconstruction framework for non-cooperative space targets based on MapAnything. First, we construct a high-fidelity dataset to inject domain-specific knowledge. Second, we introduce a SatMap-Adapter module with hierarchical sampling to enhance inference in reflective and texture-less regions. Finally, we employ Weight-Decomposed Low-Rank Adaptation (DoRA) to achieve parameter-efficient fine-tuning while freezing the backbone, significantly reducing computational costs.

The main contributions of this paper are summarized as follows:

- **Construction of a Space-Specific Dataset:** We established a multi-modal dataset featuring specular and weak-texture characteristics, effectively alleviating the data scarcity in space scenarios.
- **Hierarchical Sampling and Fusion-Aware Mechanism:** We design a fusion module that incorporates hierarchical cascade sampling and lightweight highlight-aware features. Through the dynamic integration of multi-level features, this module enhances the geometric reconstruction completeness and accuracy in regions with strong reflections and weak textures.
- **Realization of Parameter-Efficient Domain Adaptation:** By leveraging DoRA for lightweight fine-tuning of MapAnything, we transformed the general vision baseline into an expert model for spatial 3D reconstruction at a minimal computational cost.

## 2. Related Work

### 2.1. Space-Specific Reconstruction Algorithms

In On-Orbit Servicing (OOS) missions, both pose estimation and 3D reconstruction are critical prerequisites. While some prior studies tackle them simultaneously, this work specifically focuses on achieving robust metric 3D reconstruction of non-cooperative targets under extreme space lighting, assuming the initial camera poses are provided by onboard navigation systems. Traditional methods relying on handcrafted features (e.g., SIFT, SfM) [1,2,3,4] often fail due to the extreme illumination contrast and specular Multi-Layer Insulation (MLI) surfaces typical of space environments. Deep learning approaches like SPN [5] and Kine-AI [6] improved pose estimation significantly but remain limited by their reliance on known CAD models. Recently, Implicit Neural Representations [7,8] have emerged to address these challenges: Sat-NeRF [9] models transient lighting effects, Spacecraft-NeRF [10] utilizes spherical harmonics for illumination decoupling, and SparseNeRF [11] handles sparse view constraints. However, these “optimization-based” expert systems generally suffer from high computational costs and sensitivity to initialization, requiring lengthy per-scene training that hinders real-time on-orbit application [12,13,14,15].

### 2.2. General Feed-Forward 3D Reconstruction Models

The field is shifting from single-scene optimization to general feed-forward prediction [16,17]. Early attempts like Pixel-NeRF [18] and Transformer-based models (LRM [19], InstantSplat [20]) achieved rapid generation but often prioritized perceptual quality over engineering-grade geometric accuracy. A milestone was reached with DUSt3R [21], which directly regresses dense 3D pointmaps, with subsequent optimizations in MASt3R [22] and VGGT [23]. MapAnything [24] represents the state-of-the-art, enabling metric reconstruction via a Global Metric Scale Factor. Despite its performance in natural scenes, MapAnything faces a severe domain gap in space. Trained on diffuse terrestrial data, it struggles with the high dynamic range (HDR) lighting and specular materials of space targets. Retaining its metric reconstruction capabilities while incorporating physics-based constraints for space environments is key to enhancing its applicability.

### 2.3. Public Datasets for Space Targets

High-quality datasets are essential for data-driven models. SPEED [25] and SPEED+ [26] established benchmarks for pose estimation using synthetic and Hardware-in-the-Loop (HIL) data, respectively. SwissCube [27] provides rare on-orbit footage for **simulation-to-reality (sim-to-real)** verification, allowing networks trained on synthetic data to be rigorously evaluated against actual space environments. Meanwhile, datasets like SPARD [28], and URSO [29] focus on neural rendering under varying illumination. However, existing datasets are insufficient for high-fidelity reconstruction; most lack dense geometric ground truth (e.g., depth maps) and oversimplify complex surface properties like MLI reflections. To bridge this gap, we introduce a novel dataset featuring HDR lighting, complex materials, and dense geometric annotations to facilitate the domain adaptation of general reconstruction models.

## 3. Method

Addressing the critical challenges of high dynamic range illumination, texture sparsity, and monotonic backgrounds in space environments, this chapter proposes a 3D reconstruction network architecture tailored for non-cooperative space targets, inspired by the VGGT paradigm.

**Figure 2:**
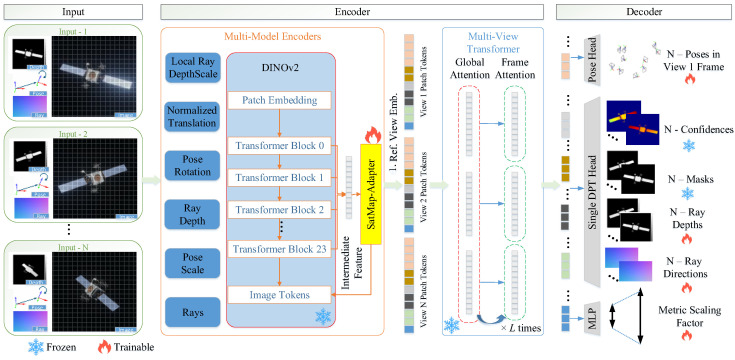
Overall architecture and fine-grained parameter freezing strategy. To minimize computational overhead, the DINOv2 encoder backbone and the MapAnything mask and confidence heads remain completely frozen. Gradient updates are strictly restricted to the newly integrated SatMap-Adapter, the attention modules, and the specific decoding branches for pose, depth, and scale estimation via DoRA adaptation.

First, we outline the MapAnything baseline framework, elucidating its principles of factorized scene representation and flexible input-output interfaces, while highlighting its efficiency advantages in resource-constrained space environments. Subsequently, to mitigate geometric degradation induced by HDR lighting, we propose a surface detail enhancement mechanism that integrates improved multi-scale feature fusion with direct photometric constraints. Finally, an efficient fine-tuning strategy based on Weight-Decomposed Low-Rank Adaptation (DoRA) is introduced, with a specific focus on its application in decoder optimization. The proposed method adopts an end-to-end architecture, and the overall pipeline is illustrated in Figure 2.

### 3.1. Overview of the Baseline Framework

MapAnything is a universal end-to-end 3D reconstruction system based on feed-forward perception [24]. It utilizes a Transformer model pre-trained on large-scale datasets to achieve direct inference from 2D images to 3D structures. Specifically, this method can recover the absolute and real physical metric scale. This capability is achieved because its network is extensively trained on massive datasets containing ground-truth 3D metric annotations, thereby implicitly learning and capturing the scale priors of the physical world. Consequently, the reconstructed 3D models possess real-world physical proportions rather than merely relative depth or size. Unlike per-scene optimization methods such as NeRF, MapAnything adopts a single-pass inference paradigm. This approach directly avoids the time-consuming iterative optimization process, significantly reducing computational latency and memory consumption. This characteristic demonstrates great application potential on resource-constrained platforms such as satellite onboard systems.

The core idea of the framework is factorized representation. It disentangles complex 3D scene reconstruction into four independently predictable components: a global metric scale factor (m, local ray directions ($R_{i}$), relative ray depths ($D_{i}$), and relative camera poses ($P_{i}=(O_{i},{\tilde{T}}_{i})$). This design greatly enhances the model’s robustness under varying input conditions. The model architecture proceeds as follows:

**(1)**  **Input Encoding and Alignment:**

The model employs a Siamese network architecture to process N views of RGB images and optional geometric priors. Image features are extracted using DINOv2 (ViT-L). Geometric inputs are decomposed into dense components (e.g., ray depths) and global components (e.g., camera poses). Dense components are encoded into patch features via a shallow convolutional encoder, while global components are mapped through an MLP and then broadcast. After normalization, the patch features for each view are element-wise summed with the broadcast global features to form unified patch tokens. Subsequently, a fixed reference view embedding is added to the features of the first view, and a single learnable scale token is appended to the set of patch tokens across all N views. These tokens are then fed into an alternating-attention transformer.

**(2)**  **Transformer Backbone:**

At the core lies a 24-layer Transformer utilizing an Alternating Attention mechanism. The network cycles between “Global Self-Attention” (facilitating multi-view fusion and epipolar geometry reasoning) and “Frame Self-Attention” (focusing on local feature enhancement and independent normalization). Additionally, a learnable Scale Token is introduced specifically to perceive the global absolute scale of the scene.

**(3)**  **Factored Decoding:**

Following feature interaction, the model outputs geometric factors in parallel through distinct prediction heads: a Dense Prediction Transformer (DPT) [30] head predicts dense ray directions and depths; a Pose head predicts quaternions and translations; and a Multi-Layer Perceptron (MLP) [31] head predicts the global scale.

Finally, the system reconstructs precise metric 3D point clouds $X_{i}^{\mathrm{metric}}$ for each input image i∈[1,N] by combining the predicted geometric factors according to the following formula:

(1) $$X_{i}^{\mathrm{metric}}=m\cdot (O_{i}\cdot (R_{i}\cdot {\tilde{D}}_{i})+{\tilde{T}}_{i})$$

where $X_{i}^{\mathrm{metric}}$ is the final metric 3D point cloud for the i-th view; m is the predicted global metric scaling factor; $R_{i}$ and ${\tilde{D}}_{i}$ are the predicted local ray directions and up-to-scale ray depths, respectively. For the relative pose terms, $O_{i}$ is the rotation matrix (derived from the predicted quaternion $Q_{i}$) and ${\tilde{T}}_{i}$ is the up-to-scale translation vector. Specifically, $O_{i}$ and ${\tilde{T}}_{i}$ represent the relative pose of image i in the coordinate frame of the first reference image. This approach enables efficient and flexible scene reconstruction while preserving metric accuracy.

### 3.2. SatMap-Adapter Perception Fusion Module

The original MapAnything framework primarily relies on semantic features extracted by DINOv2 for geometry inference, demonstrating strong reconstruction performance in conventional ground-level scenes [32]. However, when applied to space environments, the model faces unique radiometric and geometric challenges: space targets (e.g., satellites) are often covered with highly reflective multi-layer insulation, solar panels, and metallic thermal surfaces, and operate under extreme high-dynamic-range lighting without atmospheric attenuation. Such strong illumination and high specular reflection tend to cause feature degradation in the semantically pre-trained DINOv2 backbone, which struggles to accurately infer surface normal, leading to holes or distortions in the reconstructed geometry.

**Figure 3:**
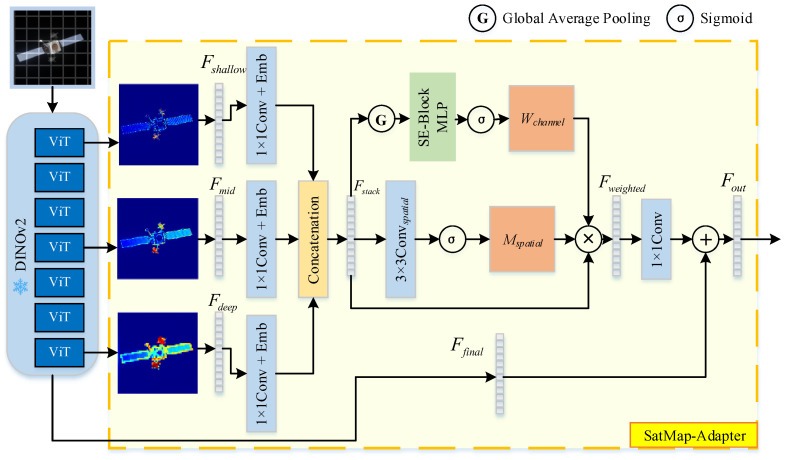
Internal structure and feature processing pipeline of the SatMap-Adapter module. This architecture employs a hierarchical cascade sampling strategy to align and reduce the dimensions of multi-level backbone features. A lightweight adaptive fusion module dynamically integrates shallow photometric cues, intermediate structural information, and deep semantic features to mitigate feature degradation caused by extreme space illumination and severe specular reflections.

To address this, we propose an enhanced architecture named SatMap-Adapter. The schematic diagram of the proposed architecture is illustrated in Figure 3. Instead of solely depending on final-layer features, this architecture adopts a hierarchical cascade sampling strategy, extracting, aligning, and reducing dimensions of multi-level backbone features to ensure compatibility with subsequent network inputs. Furthermore, we introduce a lightweight Illumination-Adaptive Feature Fusion Module that dynamically integrates high-frequency photometric cues from shallow layers, local structural information from intermediate layers, and semantic-topological features from deep layers, thereby significantly improving the geometric reconstruction accuracy of MapAnything under complex space illumination conditions.

**(1)**  **Hierarchical Feature Extraction**

The original MapAnything relies on semantic features extracted by DINOv2 for geometry inference. Although DINOv2 exhibits strong semantic understanding, its ViT-based architecture actively suppresses high-frequency variations in deeper layers—particularly in the final layers of ViT-L—resulting in the excessive smoothing of thin satellite structures such as antennas and trusses during reconstruction. While this characteristic benefits natural image classification, it becomes a critical limitation in satellite 3D reconstruction. Specular highlights on satellite surfaces, which are determined jointly by light direction, view direction, and surface normal, constitute strong geometric cues. The suppression of highlight information in DINOv2’s deep features leads to the loss of key signals necessary for surface normal inference when MapAnything solely depends on final-layer outputs.

To address this, the core design of SatMap-Adapter introduces a bypass network that extracts multi-level features from DINOv2 to construct a feature pyramid, spanning from low-level photometric cues to high-level semantic abstraction. To comprehensively capture the hierarchical progression of features, we propose a layer-wise extraction strategy—selecting blocks at either uniform or log-spaced depth intervals—to obtain the following four feature levels:

- Shallow features $F_{shallow}$: Focus on edges, texture gradients, and high-frequency illumination variations, capable of retaining specular reflection information;
- Middle features $F_{mid}$: Correspond to part-level representations, enabling differentiation between structures such as solar panels and the main body, and supporting local geometric smoothing constraints;
- Deep features $F_{deep}$: Contain high-level semantic information before excessive smoothing occurs;
- Final features $F_{final}$: The final-layer features originally used by MapAnything, providing global semantic consistency and ensuring topologically plausible reconstruction.

**(2)**  **Feature Alignment and Position Encoding**

Directly concatenating intermediate features from DINOv2 ViT-L would impose a substantial computational burden on the subsequent MapAnything pipeline. To address this, a lightweight dimensionality reduction mapping is designed.

Specifically, we utilize a hook mechanism to extract raw feature maps $F_{k}\in {\mathbb{R}}^{H\times W\times D}$ from *k*-th layers of the encoder. To mitigate the computational overhead while aligning the feature dimensions, we employ a convolution ${\mathrm{Conv}}_{1\times 1}^{k}$ to project the channel dimension of the feature maps to a unified size of (e.g., 256). Furthermore, to explicitly preserve the hierarchical information, we incorporate a learnable layer embedding $E_{level}^{k}$, which informs the network of the specific layer origin of the features. Consequently, the processed feature for a given layer can be formulated as follows:

(2) $$P_{k}={\mathrm{Conv}}_{1\times 1}^{k}(F_{k})+E_{level}^{k}$$

The aligned features from different layers are then concatenated along the channel dimension:

(3) $$F_{stack}=\mathrm{Concat}([P_{k}])$$

where $F_{stack}\in {\mathbb{R}}^{H\times W\times (k\times D_{enbed})}$.

**(3)**  **Illumination-Adaptive Feature Fusion Module**

As a pivotal component of the SatMap-Adapter, the Illumination-Adaptive Feature Fusion Module (IAFFM) addresses the limitations of standard feature aggregation in high-contrast space environments. Unlike inverse rendering approaches that explicitly solve for physical reflectance properties via BRDF or microfacet formulations, IAFFM adopts a data-driven heuristic paradigm to implicitly capture the geometric cues embedded within specular highlights.

Although specular highlights are physical phenomena governed jointly by light direction, view direction, and surface normals, they inherently embed valuable geometric and material cues. Our method extracts these cues in a purely data-driven manner without explicit physical modeling. We observe that shallow network layers effectively retain high-frequency photometric intensities and texture gradients that are crucial for localizing specular boundaries. Conversely, deep layers capture robust semantic context but tend to over-smooth these sharp geometric features. Given that naive feature concatenation is inherently linear and ill-suited for mitigating non-linear specular interferences, IAFFM employs an attention mechanism to dynamically reweight and fuse these multi-level features. Consequently, the module adaptively assigns higher weights to shallow layers in regions exhibiting strong specular reflections to leverage the preserved high-frequency gradients for precise surface normal inference. In texture-rich diffuse regions, the model prioritizes deep semantic features.

In terms of implementation, the aligned multi-scale feature tensor $F_{stack}$ is first processed by a lightweight convolutional network to generate a spatial weight map $M_{spatial}$.

(4) $$M_{spatial}=\sigma ({\mathrm{Conv}}_{spatial}(F_{stack}))$$

where σ denotes the Sigmoid activation function, which constrains the generated weights strictly within the interval [0,1] to ensure normalization. ${\mathrm{Conv}}_{spatial}$ is designed as a lightweight convolutional sub-network tasked with identifying pixels that exhibit texture gradient anomalies (such as specular reflection boundaries) by capturing local contextual information.

This map functions as an implicit “Highlight Mask,” quantifying the information density of each pixel and identifying regions subject to drastic illumination variations. Upon detecting such anomalies, the network outputs a high response value (approaching 1), thereby explicitly guiding the subsequent fusion module to prioritize these spatial locations for adaptive processing.

Subsequently, a channel weight $W_{channel}$ is generated utilizing the SE-Block mechanism [33], aimed at dynamically recalibrating the importance of cross-level features based on global context. Specifically, Global Average Pooling (GAP) is applied to $F_{stack}$, followed by an MLP to model non-linear channel correlations, and finally normalized via a Sigmoid activation function.

(5) $$W_{channel}=\sigma (\mathrm{MLP}(\mathrm{GlobalAvgPool}(F_{stack})))$$

This mechanism endows the network with adaptive regulation capabilities: it prioritizes deep semantic features in uniformly illuminated scenes while emphasizing shallow photometric cues in high-contrast environments. The Weighted Fusion is then performed by applying both spatial and channel weights to the feature stack:

(6) $$F_{weighted}=F_{stack}\cdot M_{spatial}\cdot w_{channel}$$

Finally, a 1×1 convolutional layer projects the fused features back to the original dimension. The output is obtained via a residual connection with the original semantic features $F_{final}$:

(7) $$F_{out}={\mathrm{Conv}}_{1\times 1}(F_{weighted})+F_{final}$$

This residual design guarantees a performance lower bound equivalent to the original MapAnything, thereby significantly enhancing training stability and convergence speed.

### 3.3. DoRA-Based Decoder Adaptation

Given that the MapAnything decoder comprises extensive high-dimensional mapping matrices, full-parameter fine-tuning is not only computationally prohibitive but also prone to compromising the pre-trained general priors. Drawing inspiration from MapSAM [34], we introduce Weight-Decomposed Low-Rank Adaptation (DoRA) to implement an efficient “freeze perception, fine-tune generation” strategy. This approach effectively mitigates catastrophic forgetting and achieves efficient task-specific adaptation while significantly reducing training overhead.

**(1)**  **Principle of DoRA**

**Figure 4:**
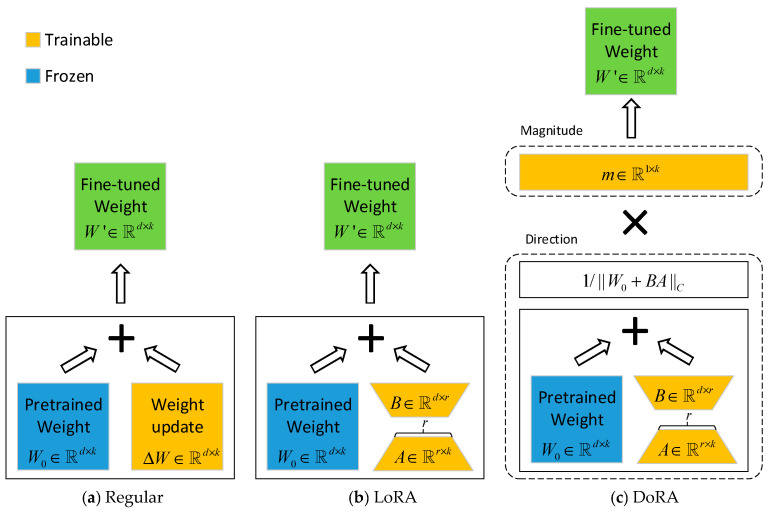
Weight update mechanisms of different fine-tuning methods.

Conventional LoRA postulates that weight updates ΔW reside within a low-rank subspace, which inherently couples changes in weight magnitude and direction. This coupling constrains the model’s fitting capacity in complex geometric regression tasks. In contrast, DoRA employs a weight normalization principle to explicitly decompose the weight matrix into a magnitude vector m and a directional matrix V [35,36]. This decoupling enables significant adjustments to feature activation intensity while preserving feature orientation, thereby ensuring semantic consistency. The difference between the two methods is shown in Figure 4. Such a mechanism is especially critical for complex space environments, as it effectively corrects scale deviations in depth estimation caused by lighting conditions. This challenge is often insurmountable for standard LoRA. Formally, the fine-tuned weight matrix W is parameterized as follows:

(8) $$W=m⊙\frac{W_{0}+BA}{{\left\|W_{0}+BA\right\|}_{c}}$$

where $W_{0}$ denotes the frozen pre-trained weights; m is the trainable magnitude vector (initialized to ${\left\|W_{0}\right\|}_{c}$); BA represents the directional increment modeled by low-rank matrices; ⊙ denotes column-wise broadcasting multiplication; and ${\left\|\cdot \right\|}_{c}$ signifies the column-wise norm, applied via column-wise broadcasting division.

**(2)**  **Targeted Optimization Strategy**

To achieve efficient fine-tuning, the DoRA module is seamlessly integrated into the key linear projection layers of the decoder’s Transformer Blocks. Specifically, for the Multi-Head Self-Attention (MHSA) and Feed-Forward Networks (FFN) in the l-th layer, the query ($W_{q}$), key ($W_{k}$), value ($W_{v}$), and output ($W_{o}$) matrices, as well as the MLP fully connected layers ($W_{fc1},W_{fc2}$), undergo DoRA-based reparameterization.

Regarding the training configuration, a rigorous fine-grained freezing strategy is adopted to decouple structural complexity from computational overhead. As shown in Figure 2, our fine-tuning strategy is as follow:

- **Fully Frozen Modules:** The heavy DINOv2 encoder backbone (approximately 300 million parameters) and the mask/confidence computation heads in the MapAnything decoder are completely frozen.
- **DoRA-Adapted Modules:** We selectively insert DoRA adapters into the attention computation modules, the newly proposed SatMap-Adapter, and the specific decoding branches responsible for pose, depth, and scale estimation.

The original MapAnything architecture comprises approximately 394 million parameters, primarily distributed across a 304.3 M parameter DINOv2 encoder, an 85.8 M parameter ViT-Base decoder, and various prediction heads. Conventional full-network fine-tuning necessitates updating a massive number of parameters, which is highly computationally and time-intensive. To drastically alleviate this computational burden while strictly preserving prediction accuracy, we employ the DoRA strategy, setting the parameters to rank r=32 and scaling factor α=32.

By freezing the heavyweight backbone and optimizing only the decoder alongside the injected low-rank matrices and specific modules, the number of trainable parameters is compressed to approximately 85 M. Compared to the 394 M parameters required for full-network fine-tuning, this constitutes a substantial reduction of 78.48%. This drastic decrease fundamentally unburdens the backward pass from massive gradient computations. Furthermore, by relieving the optimizer from storing momentum and variance states for hundreds of millions of frozen parameters, this strategy significantly reduces VRAM consumption. Ultimately, this lightweight adaptation ensures excellent numerical stability, facilitates rapid convergence on limited synthetic data, and maintains high precision in real-world space imagery generation, thereby significantly mitigating the risk of overfitting in few-shot scenarios.

**(3)**  **Train Loss**

To optimize the proposed network end-to-end, we strictly adopt the comprehensive loss formulation from the original MapAnything framework. The total loss L is a carefully balanced weighted sum of multiple geometric, scale, and detail constraints, defined as follows:

(9) $$L=10\cdot L_{\mathrm{pointmap}}+L_{\mathrm{rays}}+L_{\mathrm{rot}}+L_{\mathrm{translation}}+L_{\mathrm{depth}}+L_{\mathrm{lpm}}+L_{\mathrm{scale}}+L_{\mathrm{normal}}+L_{\mathrm{GM}}+0.1\cdot L_{\mathrm{mask}}$$

where each term specifically regularizes a corresponding output from the distinct prediction heads:

- **Scale-Independent Losses:** $L_{\mathrm{rays}}$ and $L_{\mathrm{rot}}$ constrain the predicted dense ray directions and camera quaternions, respectively. Since rotation and ray direction are independent of scene scale, these are evaluated directly against the ground truth using angular and distance metrics.
- **Up-to-Scale Geometry Losses:** To ensure robust convergence of the spatial structure, scale-invariant losses are applied to the translation vectors ($L_{\mathrm{translation}}$), ray depths ($L_{\mathrm{depth}}$), local pointmaps ($L_{\mathrm{lpm}}$), and global world-frame pointmaps ($L_{\mathrm{pointmap}}$). Following standard practices, these regressions are computed in log-space, with $L_{\mathrm{pointmap}}$ acting as a confidence-weighted loss.
- **Global Metric Scale Loss:** $L_{\mathrm{scale}}$ penalizes the deviation between the predicted global scaling factor and the ground-truth metric scale, forcing the network to recover the absolute physical dimensions of the non-cooperative targets.
- **Detail and Mask Constraints:** Leveraging our high-fidelity synthetic dataset, we apply a normal loss ($L_{\mathrm{normal}}$) and a multi-scale gradient matching loss ($L_{\mathrm{GM}}$) to explicitly capture the high-frequency structural details of the spacecraft (e.g., antennas and solar panels). Lastly, $L_{\mathrm{mask}}$ utilizes binary cross-entropy to supervise the valid foreground masks.

For all regression terms, an adaptive robust loss mechanism is employed to mitigate the influence of potential outliers caused by complex space lighting conditions during the training process.

## 4. Datasets Construction for Space Targets

To address the limitations of existing public datasets in adapting to space environments, this section delineates the methodology employed for dataset construction. We established a hybrid dataset incorporating High Dynamic Range (HDR) illumination and complex material properties, comprising both synthetic generation and real-world acquisition. A detailed comparison between the proposed dataset and other prevailing benchmarks is presented in the Table 1.

As shown in the comparison in the above table, our dataset outperforms others in terms of the number of target categories, the complexity of target materials, and the diversity of lighting conditions. Moreover, it provides additional annotations such as mask, depth, and pose, which not only enhance the accuracy of ground truth and the variety of illumination, but also improve the dataset’s representativeness and practicality, demonstrating strong research potential.

### 4.1. Synthetic Image Generation

To construct high-fidelity simulation scenarios, we acquired a diverse array of typical spacecraft 3D models from public NASA [37] and ESA [38] databases, standardizing them into OBJ format for integration into the Blender rendering engine.

**Figure 5:**
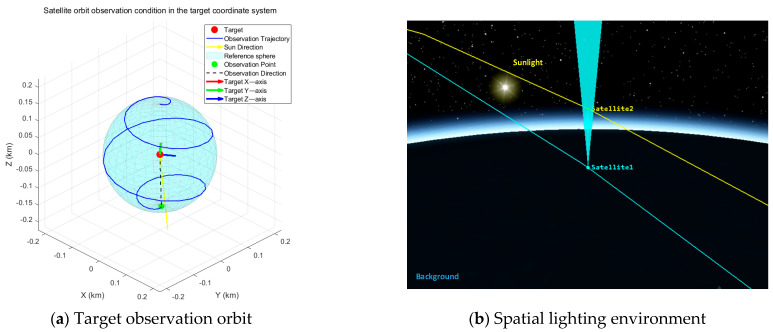
Schematic diagram of the simulated orbital and radiation environment.

Regarding illumination simulation, we placed a specific emphasis on replicating the High Dynamic Range (HDR) characteristics unique to the space environment. This involved precise modeling of Earth albedo, intense direct solar radiation, and deep shadows against the dark space background, ensuring the dataset encompasses extreme lighting conditions where specular saturation and pitch-black shadows coexist. Furthermore, to accommodate the adaptability requirements of on-orbit environments, we utilized STK12 software to simulate various complex relative motion trajectories, including approach, fly-around, and departure maneuvers. The schematic diagrams of some simulated orbits and radiation environments are presented in Figure 5. This diversity in orbital and operational configurations not only supports high-precision 3D reconstruction tasks but also provides robust data inputs for algorithms in broader aerospace domains.

**Figure 6:**
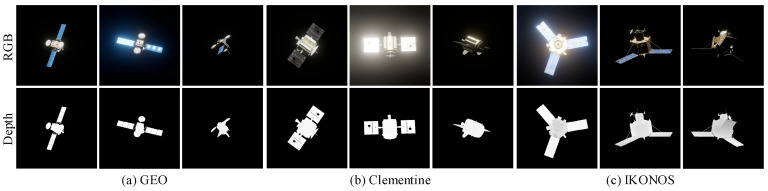
The spatial target simulation image data in our dataset.

Following scene configuration, high-fidelity imaging simulations were conducted via ray tracing based on an ideal pinhole camera model, synchronously recording camera intrinsics, extrinsics, and auxiliary masks. The underlying simulation algorithms and image quality assessment protocols are detailed in our previous work [39]. Over 50 targets were simulated in total; representative rendered images and their corresponding depth maps are illustrated in Figure 6.

### 4.2. Real Image Acquisition

**Figure 7:**
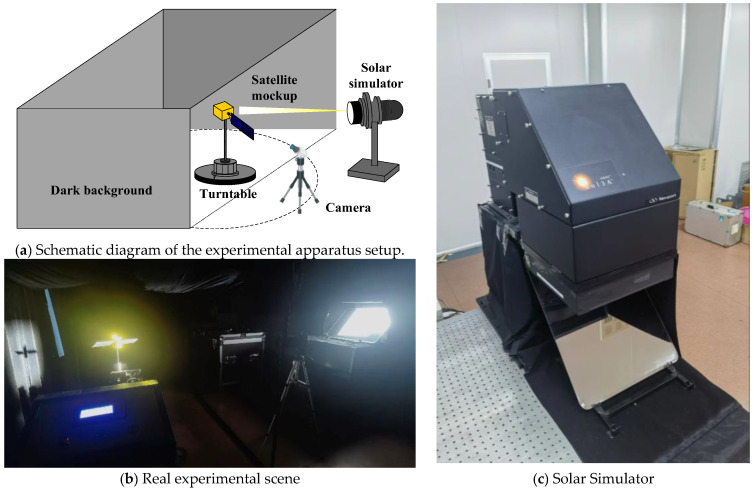
Ground experiment scene.

To bridge the domain gap between synthetic data and real-world scenarios, a physical acquisition platform was established within a laboratory darkroom. The ground experiment scene is shown in Figure 7. Multi-view image sequences of scaled satellite models were captured under realistic illumination to evaluate the model’s generalization capabilities.

The solar simulator used in this experiment is Newport Oriel Sol3A Model 94123A, and the performance parameters are shown in Table 2.

Prior to image acquisition, the camera was rigorously calibrated utilizing Zhang’s method to obtain highly accurate intrinsic parameters. This procedure provides essential geometric inputs for all subsequent testing phases. The calibration process successfully determined the camera focal length, principal point, and distortion coefficients, yielding a root mean square reprojection error of 5.6 pixels. This specific error metric quantifies the measurement uncertainty of our imaging system. The detailed calibrated camera parameters are presented in Table 3.

**Figure 8:**
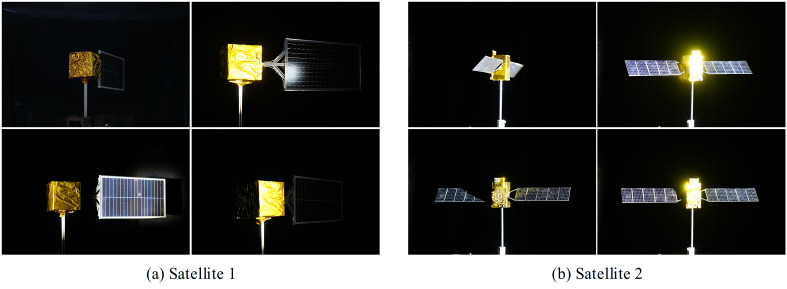
The real collected image data in our dataset.

Real-world measurement data from multiple satellite models were collected in this study, and representative data samples selected from them are presented in Figure 8.

## 5. Experiment and Analysis

### 5.1. Experimental Settings

Experiments were conducted on our custom satellite dataset, which is characterized by intense specular reflections. To ensure rigorous statistical validation, the dataset was randomly partitioned into training and testing sets using a balanced 80:20 ratio. Furthermore, to account for data variability, all experiments were independently repeated three times using different random seeds, with all quantitative metrics reported as the mean and standard deviation. All training and evaluation procedures were executed on a single NVIDIA A100 GPU. To validate the efficacy of our approach, we benchmarked it against current state-of-the-art (SOTA) feed-forward 3D reconstruction and pose estimation foundation models, including MapAnything (the baseline), VGGT, and DUSt3R.

Following standard protocols in 3D vision, we employed three categories of metrics to comprehensively assess model performance in high-reflection space scenarios:

- AbsRel (Absolute Relative Error): Measures the absolute relative deviation between predicted and ground truth depths; lower values indicate better performance.
- RMSE (Root Mean Squared Error): Quantifies the global deviation of depth values; lower is better.
- *δ* (Threshold Accuracy): The percentage of pixels where the ratio between the predicted and ground truth; higher is better.
- CD (Chamfer Distance): Evaluates the geometric structural alignment between the reconstructed point cloud and the Ground Truth (GT); lower values denote higher geometric fidelity.
- ATE (Absolute Trajectory Error): Directly measures the translational drift of camera poses; lower is better.
- Inference Time: The duration of each reconstruction inference is documented to directly reflect the model’s real-time processing capability.

### 5.2. Comparison with Baselines

The following Table 4 presents the quantitative comparison results between our method and the baselines—MapAnything, VGGT, and DUSt3R—on the self-constructed test set.

Driven by the specialized feature enhancement mechanism tailored for high reflection regions, the proposed approach achieves significant improvements in geometric reconstruction accuracy. Specifically, the absolute relative error (AbsRel) and Chamfer distance (CD) decrease by 15.23% and 20.02% respectively compared to the baseline MapAnything model. Conventional methods typically exhibit substantial depth noise in areas characterized by intense illumination and metallic surfaces. Conversely, our model successfully maintains high structural integrity under these challenging conditions. Furthermore, the proposed method achieves superior inference speeds and retains high precision. This exceptional computational efficiency validates the engineering feasibility of our architecture for on-orbit tasks and large-scale satellite data processing.

**Figure 9:**
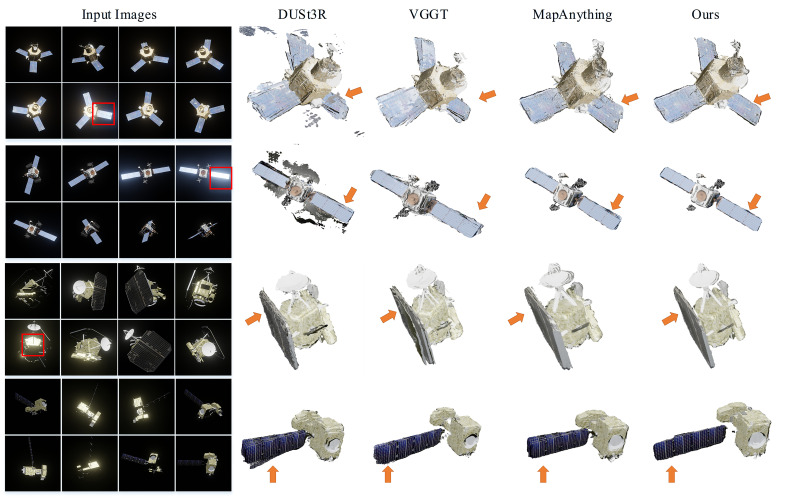
Qualitative comparisons with other methods when using different images as input. The red box indicates regions with significant lighting variations, while the arrows point to areas with the greatest differences in reconstruction quality.

Figure 9 illustrates a qualitative reconstruction comparison between the proposed method and baseline approaches in typical satellite scenarios.

As highlighted in the red bounding boxes, baseline methods predominantly misinterpret specular highlights on solar arrays and metallic surfaces as drastic depth discontinuities, resulting in severe geometric distortions and positional errors in the point cloud. In contrast, our approach, leveraged by the specular-aware fusion mechanism, effectively rectifies these artifacts to recover smooth and continuous surface topologies. Furthermore, the proposed method demonstrates superior multi-view consistency under challenging conditions involving large baselines and limited feature correspondences, yielding 3D point clouds characterized by significantly higher density and sharper structural details.

### 5.3. Ablation Studies

To validate the efficacy of the proposed components—specifically the SatMap-Adapter and the DoRA-based optimization strategy—we conducted step-wise ablation studies. The experiments followed a strict progression: (1) the original MapAnything model as the Baseline; (2) the Baseline integrated with our SatMap-Adapter; and (3) the full model further incorporating the DoRA module for acceleration. To ensure a completely fair comparison, all three configurations were trained and evaluated under identically unified hardware environments and the newly re-partitioned dataset. The detailed quantitative results are summarized in Table 5.

**Effectiveness of the SatMap-Adapter:** As detailed in the table, the standalone incorporation of the SatMap-Adapter yields a substantial reduction in depth prediction errors within high-reflection regions. The AbsRel metric decreases from 0.115 (Baseline) to 0.0911, and the Chamfer Distance (CD) drops from 1.12 to 0.95. This substantiates that the module effectively mitigates the non-linear interference of specular reflections on geometric inference through adaptive feature weighting, thereby enhancing model robustness against complex material properties.

**Advantages of DoRA Optimization:** Advantages of DoRA Optimization: As demonstrated in Table 4, the integration of DoRA resolves the apparent paradox between structural complexity and training efficiency. While the baseline adapter model requires updating the entire 394 M-parameter decoder—consuming 22.6 GB of peak VRAM and 79.5 h of training—our DoRA-based strategy strictly freezes these base weights. By optimizing only the injected low-rank matrices, the trainable parameter space is compressed to a mere 85 M. This 78.5% reduction fundamentally unburdens the backward pass from massive gradient computations and relieves the optimizer from storing extensive state variables. Consequently, peak VRAM usage drops drastically to 5.9 GB, and overall training time is significantly accelerated to 58.4 h. Ultimately, this lightweight adaptation ensures excellent numerical stability, facilitates rapid convergence, and maintains high precision in generating real-world space imagery, effectively mitigating overfitting in few-shot scenarios.

### 5.4. Generalization Assessment Experiment

**Figure 10:**
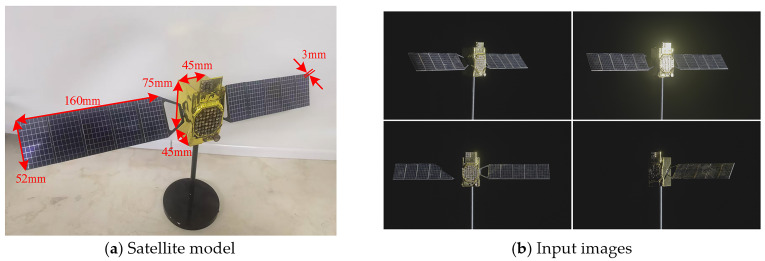
Input for the generalization assessment experiment.

To evaluate the disparity in generalization performance between the proposed improved model and the original MapAnything, the trained models were directly deployed for inference on unseen real-world satellite imagery. The target’s actual physical dimensions served as the quantitative baseline, with detailed geometric parameters and material properties provided in Figure 10 and Table 6.

To ensure precise metric scaling, the 3D reconstruction was executed by feeding both the acquired multi-view images and the calibrated camera intrinsic parameters into the network. This flexible input strategy leverages the core capabilities of the MapAnything framework to accurately anchor the estimated camera poses and dense point clouds to the absolute metric scale. Subsequently, the mean distance error between the reconstructed point cloud and the ground truth reference model was calculated as the primary quantitative evaluation metric.

**Figure 11:**
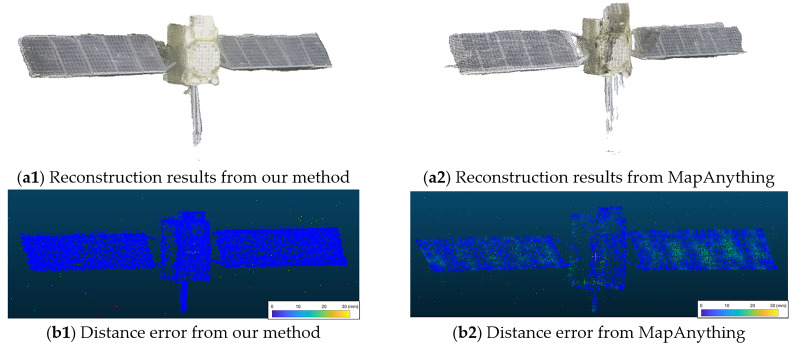
Qualitative comparison of reconstruction results.

**Figure 12:**
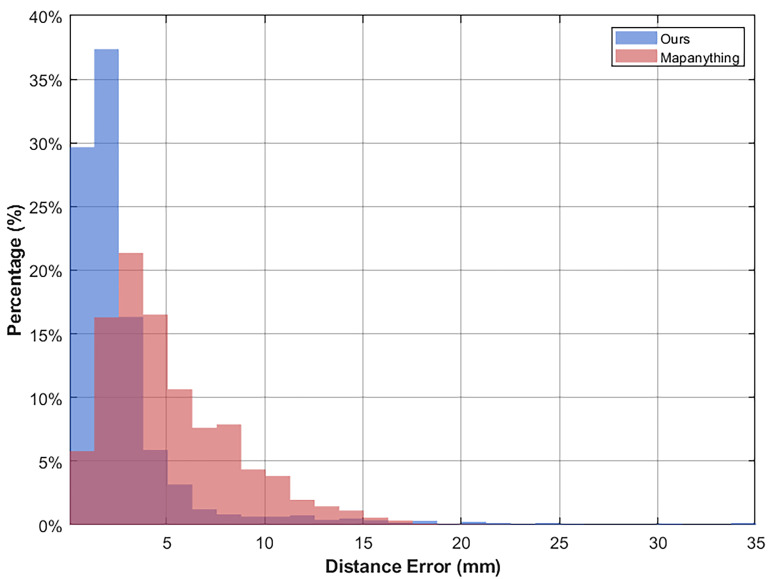
Comparison of Distance Error Distributions.

Figure 11 and Figure 12 present the qualitative and quantitative experimental results respectively. Qualitative evaluations confirm that the proposed improved model effectively mitigates geometric distortions induced by complex illumination, exhibiting superior robustness and adaptability in non-ideal imaging environments. Quantitatively, the baseline MapAnything model yields an average distance error of 5.149 mm. In contrast, our improved model significantly reduces this error to 2.824 mm.

This performance gap indicates that the original MapAnything framework struggles with unseen samples featuring strong reflective textures, which invariably leads to substantial geometric distortions. Conversely, the implemented DoRA fine-tuning strategy successfully adapts to the unique feature distributions of highly reflective space targets. It achieves this precise adaptation while maximally preserving the generic visual priors embedded within the pre-trained backbone model. Ultimately, this optimized balance delivers substantially higher geometric accuracy in practical 3D reconstruction tasks.

## 6. Conclusions and Discussion

To address the challenge of limited 3D reconstruction accuracy for non-cooperative space targets under extreme illumination conditions, this study proposes an enhanced framework based on MapAnything. By integrating a SatMap-Adapter module with the Weight-Decomposed Low-Rank Adaptation (DoRA) optimization strategy, we effectively mitigate the loss of geometric details caused by strong specular reflections on satellite surfaces. Experimental results demonstrate that the proposed method not only significantly suppresses geometric noise induced by highlights and specular effects but also achieves reconstruction accuracy superior to existing large-model baselines, all while maintaining efficient inference speeds.

This work validates the efficacy of parameter-efficient fine-tuning (PEFT) in successfully transferring the powerful representational capabilities of general vision foundation models to the challenging domain of space perception. This strategy endows the system with exceptional data adaptability and scalability; facing novel scenarios or emerging tasks, rapid model iteration can be achieved through fine-tuning with incremental data, thereby circumventing the prohibitive computational costs of full retraining. Future work will focus on extending the model’s dynamic perception capabilities, specifically targeting dynamic object tracking and reconstruction under extreme motion blur, to further meet the rigorous demands of complex on-orbit missions.

## Figures and Tables

**Table 1:** Comparison of spacecraft datasets.

Dataset	Modality	Targets	Lighting Condition	Data Supplementation	Material Complexity
SwissCube	Syn	1	Variable	Pose + Mask	Medium
SPEED+	Syn + Real	1	Variable	Pose Only	Low
URSO	Syn	2	Variable	Pose Only	Low
SPARK	Syn + Real	1	Simple	Pose + Model	Medium
Ours	Syn + Real	50+	Variable	Mask + Depth + Model	High (MLI/Wrinkles)

**Table 2:** Performance indexes of Solar Simulator.

Parameters	Value
Illumination (mm^2^)	305 × 305
Maximum angle of incidence (°)	<±0.5°
Typical power output (mW/cm^2^)	100 (1SUN), ±20% Adjustable
Uniformity	<±2%
Spectral match	9.7–16.1% (800–900 nm)

**Table 3:** Calibrated camera intrinsic and distortion parameters.

Parameter Category	Parameter Symbol	Calibrated Value
Image Resolution	Width × Height	1920 × 1080
Focal Length	$f_{X}$	1450.25
	$f_{Y}$	1451.10
Principal Point	$c_{x}$	965.50
	$c_{y}$	542.30
Radial Distortion	$k_{1}$	−0.1254
	$k_{2}$	0.0842
	$k_{3}$	−0.0021
Tangential Distortion	$p_{1}$	0.0015
	$p_{2}$	−0.0008
Reprojection Error	RMSE	5.6 pixels

**Table 4:** Reconstruction quality results compared with other methods.

Method	AbsRel ↓	RMSE ↓	δ ↑	ATE ↓	CD ↓	Time ↓
DUSt3R	0.112 ± 0.005	0.456 ± 0.012	0.854 ± 0.008	0.045 ± 0.003	1.23 ± 0.04	9.7 s
VGGT	0.135 ± 0.006	0.512 ± 0.015	0.810 ± 0.009	0.052 ± 0.004	1.45 ± 0.05	3.2 s
MapAnything	0.105 ± 0.004	0.420 ± 0.010	0.885 ± 0.006	0.038 ± 0.002	1.10 ± 0.03	2.1 s
Ours	0.089 ± 0.002	0.350 ± 0.008	0.920 ± 0.004	0.032 ± 0.001	0.88 ± 0.02	2.2 s

**Table 5:** Results of Ablation Experiments.

Model Setting	SatMap-Adapter	DoRA	AbsRel ↓	CD ↓	Trainable Params	Peak VRAM	Training Time
Baseline (MapAnything)	×	×	0.115 ± 0.005	1.12 ± 0.04	390 M	22.3 G	77.6 h
Baseline + SatMap-Adapter	√	×	0.091 ± 0.003	0.95 ± 0.03	394 M	22.6 G	79.5 h
Ours (Full)	√	√	0.089 ± 0.002	0.88 ± 0.02	85 M	5.9 G	58.4 h

**Table 6:** Satellite model size and material.

Parameters	Value
Satellite body size (mm)	45 × 45 × 75
Satellite panel size (mm)	160 × 52 × 3
Satellite body material	Gold polyimide film
Satellite panel material	Monocrystalline silicon cell

## Data Availability

The data presented in this study are available on request from the corresponding author.
